# All-polyethylene versus metal-backed tibial components in total knee arthroplasty: a meta-analysis of randomized controlled trials

**DOI:** 10.1007/s00590-023-03594-y

**Published:** 2023-05-30

**Authors:** Aissam Elmhiregh, Yousef Abuodeh, Osama Alzobi, Bashir Zikria, Mohd Alkhayarin, Bernard F. Morrey

**Affiliations:** 1 Almowasafat Hospital, Tripoli, Libya; 2 Department of Orthopaedic Surgery, Surgical Specialty Center, Hamad Medical Corporation, Doha, Qatar; 3 Aspetar Orthopaedic and Sports Medicine Hospital, Doha, Qatar; 4 Mayo Clinic, Rochester, MN USA

**Keywords:** Total knee arthroplasty, TKA, All-polyethylene, Metal-backed, RSA, Knee society score

## Abstract

**Objectives:**

The design of tibial trays for total knee arthroplasty (TKA) has been a topic of research for several decades. Although all-polyethylene trays were developed to address issues such as osteolysis and to enhance the longevity of the prosthesis, as well as knee range of motion, metal-backed designs have remained the most commonly used type of prosthesis. This meta-analysis aimed to compare the clinical, radiological, and survival outcomes of both designs.

**Methods:**

Five databases were searched from inception until October 1, 2020, for randomized controlled trials (RCTs) that compared the outcomes of all-polyethylene and metal-backed tibial components in TKA. The outcomes of interest included range of motion, knee society score, stairs climbing scores, radiostereographic analysis, survivorship and complication. This review was conducted in line with PRISMA (Preferred Reporting Items for Systematic Reviews and Meta-Analyses) guidelines. Risk of bias was assessed using the Newcastle–Ottawa tool.

**Results:**

A total of 14 RCTs with 1367 TKA were included with a mean age of – years and – years for all-polyethylene and metal-backed tibial components groups, respectively. All-polyethylene group demonstrated statistically significant differences in five-year survivorship (OR 0.27; 95% CI 0.10–0.75; *p *value 0.01) and stairs climbing score (OR − 2.07; 95% CI − 3.27–0.87; *p *value 0.0007) when compared to the metal-backed group. The metal-backed design was significantly more radiographically stable in anterior–posterior, varus–valgus, and internal–external rotations at the 2-year follow-up compared to all-polyethylene tibias (OR − 0.09; 95% CI − 0.16 to − 0.02; *p* value 0.02) as per the pooled radiostereographic analysis. However, ten-year survivorship (OR 0.92; 95% CI 0.53–1.60; *p* value 0.78), range of motion (OR − 0.57; 95% CI − 2.00–0.85, *p* value 0.43), knee society scores (OR 1.38; 95% CI − 0.47–3.23, *p* value 0.14), and complications (OR 0.83; 95% CI 0.5–1.39, *p* value 0.48) were comparable between both groups.

**Conclusions:**

While this meta-analysis suggests that all-polyethylene tibial components in total knee arthroplasty may offer advantages over metal-backed components in terms of five-year survivorship, and stairs climbing score, this finding should be considered in the context of potential confounding factors. Nonetheless, based on the results, the all-polyethylene implant should be considered a viable choice for primary knee replacement.

**Level of evidence:**

I.

## Introduction

Metal-backed tibial components have been the preferred implant design in knee replacement surgeries over the all-polyethylene counterpart since their introduction in the 1970s. The first generations of all-polyethylene tibias were hindered by drawbacks such as osteolysis and earlier implant failure, leading to decreased popularity among arthroplasty surgeons over the last few decades [1, 2]. However, the theoretical value of modularity made the metal-backed option more attractive.

The all-polyethylene tibias used in TKA are renowned for their reliability, minimal bone resection requirements, and lack of implant migration and backside wear. Furthermore, the all-polyethylene design is significantly more cost-effective than the metal-backed alternative [3, 4]. This is particularly advantageous given the increasing demand for knee replacements worldwide, with the potential to reduce implant costs by up to 50% [5, 6]. Recent advances in all-polyethylene tibial components have demonstrated equivalent clinical reliability when compared to metal-backed designs [2].

The concept of modularity in metal-backed tibial components is appealing, as evidenced by several biomechanical studies showing its theoretical advantages in load distribution and resistance to implant failure [7, 8]. Additionally, the ability to remove the polyethylene liner without affecting the tibial fixation simplifies revision for bearing wear. There is also the potential for improved motion with reduced thickness or greater stability with a thicker insert. However, despite these theoretical benefits, none have been shown to have clinical significance. In contrast, drawbacks of metal-backed tibial components include backside wear caused by micromotion at the polyethylene–metal interface, and the need for a larger bone cut to accommodate the metal tray at the expense of a thinner polyethylene liner [9, 10].

The purpose of this study was to present the most comprehensive evidence comparing all-polyethylene and metal-backed tibial components in total knee arthroplasty. We evaluated various clinical and radiological variables from the studies included in our analysis. We hypothesized that there would be no significant difference between the two groups in terms of survivorship, functional outcomes, and complication rates.

## Materials and methods

This meta-analysis was performed according to the Preferred Reporting Items for Systematic Reviews and Meta-Analyses (PRISMA) guidelines, with a PRISMA checklist and algorithm [11].

### Search strategy

PubMed/Medline, CINAHL, Cochrane, Embase, and Google Scholar databases were systematically searched from inception until October 1, 2020, to identify articles in peer-reviewed journals. The search was performed using the following keywords and their derivatives: "Knee arthroplasty," "Knee replacement," "Joint replacement," "Total knee," "Tibial," "All-polyethylene," "Metal-backed," "Tibail tray," "Randomized," and "RCT." Search results were screened against the eligibility criteria by two authors independently based on the title and/or abstract. Conflicts were resolved via a discrepancy meeting with a third, more senior author. A full-text review of articles that met the eligibility criteria was performed, and references of included articles were manually sought to ensure all relevant studies were included.

### Outcomes of interest

The study evaluated several outcomes of interest, including range of motion, clinical and functional knee society scores at the final follow-up, stair climbing scores at one and five years, survivorship at five and ten years, and overall complication rates. Radiostereographic analysis was pooled at two years, encompassing anterior–posterior rotation, internal–external rotation migration, and varus–valgus malalignment.

### Eligibility criteria

#### Inclusion criteria

1. All original comparative level I of evidence RCTs reporting primary TKA indicated in all-polyethylene versus metal-backed tibial components.
2. Studies with a minimum follow-up period of one year.
3. RCTs that published complete manuscript with available data in English.
4. RCTs that published clear outcome measures with attached data presented as or can be transferred to mean and standard deviation values.

#### Exclusion criteria

1. Non-comparative or not reporting outcomes.
2. Review articles, cross-sectional, case series and reports.
3. Preclinical or animal studies.
4. Studies with incomplete or unextractable data.
5. Studies published in languages other than English.

### Data extraction methods

A pre-designed data collection sheet in Microsoft Excel was utilized by two independent reviewers to extract data. The extracted data included demographic information such as the first authors’ surnames, study year, design and location, the mean age of patients, number of participants, and mean follow-up period. Other information collected included whether patellar resurfacing was performed, the type of prosthesis used, range of motion, clinical and functional knee society scores, stair climbing scores, survivorship, overall complication rates, and radiostereographic analysis.

### Qualitative assessment (risk of bias)

Two authors assessed the methodological quality of the included studies using the Newcastle–Ottawa tool, which comprises of three key domains; patient selection, comparability, and outcomes [12]. A higher overall score indicates a lower risk of bias; a score of 5 or less (out of 9) corresponds to a high risk of bias.

### Quantitative analysis

The meta-analysis was conducted using RevMan V.5.0.18.33 (The Cochrane Collaboration, Copenhagen, Denmark). Continuous variables were presented as mean and standard deviation, or standardized using validated formulas [13] when presented as range, confidence interval, or first and third interquartile. Studies that could not be standardized were excluded [14]. For four studies with graph data, the Digitalizer application was used. Dichotomous variables were analyzed using Relative risk (RR) with 95% CI. Heterogeneity was measured using I2, and results were considered statistically significant at *p* < 0.05.

## Results

### Study selection

**Fig. 1:**
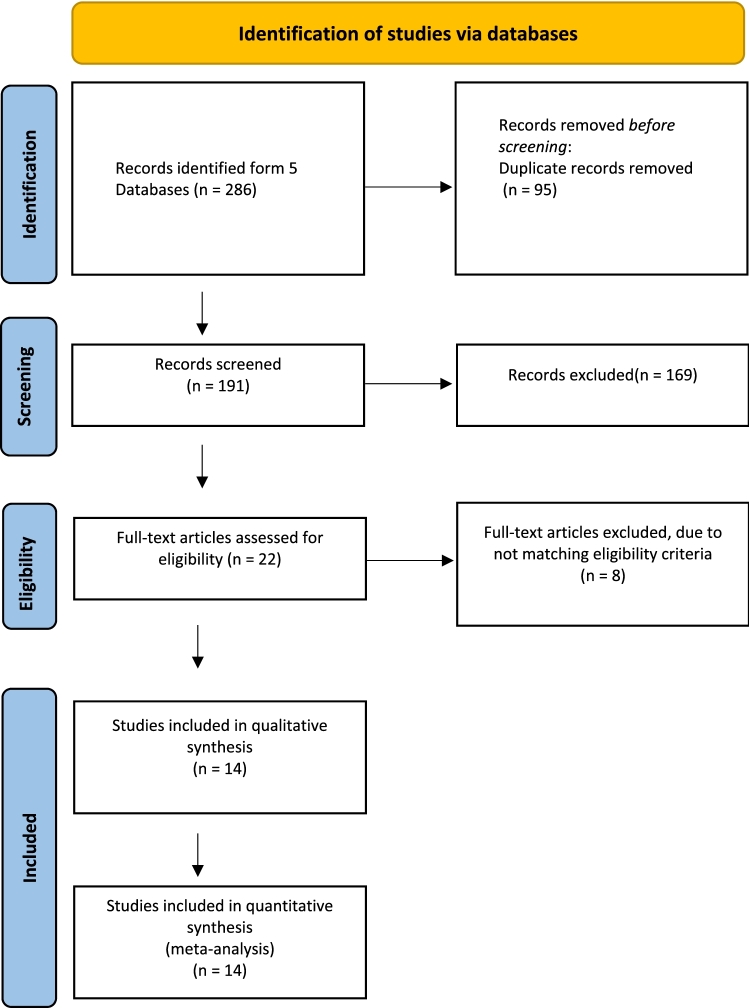
PRISMA checklist and algorithm

Searching the databases yielded 286 articles, and after removing 95 duplicates, 191 records were screened by title and abstracts, of which 169 were excluded. A total of 22 papers were eligible for a full-text review. As a result, 14 studies met the eligibility criteria and were included in the qualitative and quantitative synthesis. The PRISMA flowchart is displayed in Fig. 1.

### Study characteristics

**Fig. 2:**
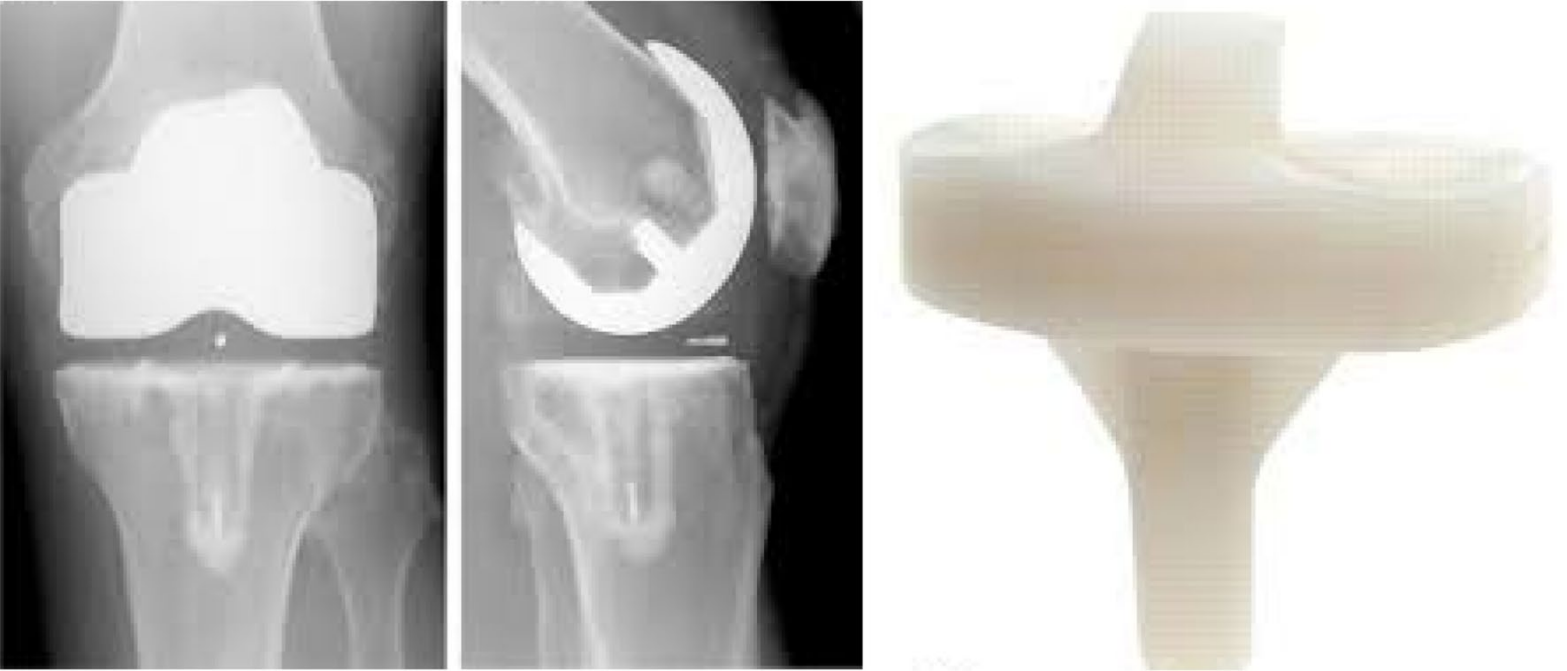
Demonstration of all-polyethylene tibial component

A total of 14 RCTs investigating the impact of all-polyethylene and metal-backed tibial components on TKR were included (Table 1). The included studies were published during the period from 2000 to 2019. The studies included in the meta-analysis recorded a total of 1367 TKA procedures, of which 686 patients received an all-polyethylene tibial tray, and the remaining 681 received a metal-backed tibial tray. All included RCTs utilized a single brand and design of prosthesis and randomized patients based on the type of tibial implant within the same brand. The randomized groups were well-matched in terms of age and gender. Follow-up duration varied among the studies, ranging from 1 to 10 years, resulting in some heterogeneity. We specifically analyzed studies with comparable follow-up durations, categorizing them as short-term (1–5 years), long-term (6–10 years), and final follow-up (1–10 years). None of the studies reported the use of vitamin E-treated polyethylene, probably because it was not well established at the time of the included studies. However, only two studies, Hamersveld et al. [15] and Bettinson et al. [16], mentioned the use of Ultra-high-molecular-weight polyethylene (Fig. 2).

**Table 1:** Characteristics of the included studies

Study	Year of publication	LoE	Number of knees	APT	MPT	Follow-up	Patellar resurfacing	Type of prosthesis
Kalisvaart [18]	2012	I	151	75	76	5 ys	YES	Posterior-stabilized knee design (P.F.C. [Press-Fit Condylar] Sigma; DePuy, Warsaw, Indiana)
Gioe [23]	2000	I	200	103	97	3 ys	YES	Cruciate retaining (Press Fit Condylar, J & J/Depuy, Warsaw, IN)
Adalberth [21]	2000	I	34	17	17	2 y	NO	Cruciate retaining AGC cemented TKA (Biomet, Warsaw, IN)
Adalberth [19]	2001	I	38	20	18	2 ys	YES	Freeman-Samuelson (Sulzer Orthopaedics AG, Zug, Switzerland) cemented TKA
Norgren [3]	2004	I	23	12	11	2 ys	NA	Profix cemented TKA (Smith & Nephew, Memphis, Tennessee, USA)
Pagnano [12]	2004	I	160	80	80	1 y	YES	posterior-stabilized knee (Sigma Press-Fit Condylar, DePuy, Warsaw, IN)
Gioe [22]	2007	I	167	97	70	10 ys	YES	Cruciate retaining (DePuy PFC, DePuy, Warsaw, IN)
Abdel [17]	2018	I	116	50	66	10 ys	YES	Posterior-stabilized (Press Fit Condylar (PFC) Sigma
Van Hamersveld [15]	2018	I	59	29	30	2 ys	NO	cruciate-retaining Triathlon (Stryker, Warsaw, USA)
Hasan [20]	2019	I	56	27	29	2 ys	Re-shapped patella	Posterior-stabilized knee Triathlon (Stryker, Warsaw, USA)
Bettinson [16]	2009	I	293	138	155	10 ys	NO	Kinemax Plus cruciate-retaining implant
Hyldahl part 1 [25]	2005	I	35	19	16	2 ys	NO	AGC total knee prosthesis (Anatomic Graduated Component; Biomet, Warsaw, IN)
Hyldahl part 2 [29]	2005	I	35	19	16	2 ys	NO	AGC total knee prosthesis (Anatomic Graduated Component; Biomet, Warsaw, IN)
Muller [8]	2006	I	40	21	19	2 ys	NA	Cruciate-retaining condylar design, PFC-∑

### Quality assessment

The scores of all 14 studies ranged from 7 to 9, indicating a low overall risk of bias. [12]. A summary of the qualitative assessment, according to the Newcastle–Ottawa scale, is shown in Table 2.

**Table 2:** Newcastle–Ottawa Scale [13]

Study	Type	Selection				Comparability	Exposure/outcome			Total number of stars
Kalisvaart [18]	RCT	⋆	⋆	⋆	⋆	⋆⋆	⋆	⋆	⋆	9
Gioe [23]	RCT	⋆	⋆	⋆	⋆	⋆⋆		⋆	⋆	8
Adalberth [21]	RCT	⋆	⋆	⋆	⋆	⋆⋆		⋆	⋆	8
Adalberth [19]	RCT	⋆	⋆	⋆	⋆	⋆⋆		⋆	⋆	8
Norgren [3]	RCT	⋆	⋆	⋆	⋆	⋆⋆		⋆	⋆	8
Pagnano [12]	RCT	⋆	⋆	⋆	⋆	⋆⋆			⋆	7
Gioe [22]	RCT	⋆	⋆	⋆	⋆	⋆⋆		⋆	⋆	8
Abdel [17]	RCT	⋆	⋆	⋆	⋆	⋆⋆	⋆	⋆	⋆	9
Van Hamersveld [15]	RCT	⋆	⋆	⋆	⋆	⋆⋆		⋆	⋆	8
Hasan [20]	RCT	⋆	⋆	⋆	⋆	⋆⋆		⋆	⋆	8
Bettinson [16]	RCT	⋆	⋆	⋆	⋆	⋆⋆		⋆	⋆	8
Hyldahl part 1 [25]	RCT	⋆	⋆	⋆	⋆	⋆⋆		⋆	⋆	8
Hyldahl part 2 [29]	RCT	⋆	⋆	⋆	⋆	⋆⋆		⋆	⋆	8
Muller	RCT	⋆	⋆	⋆	⋆	⋆⋆		⋆	⋆	8

### Functional knee society score

**Fig. 3:**
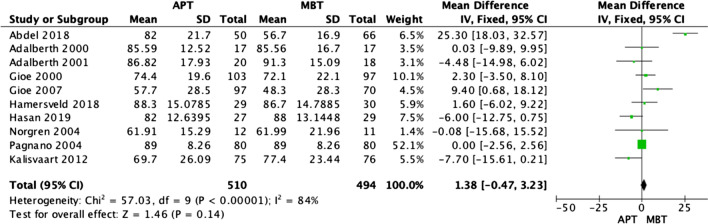
Forest plot of functional knee society score at the final follow-up between all-polyethylene (APT) and metal-backed (MBT) tibias, CI: confidence interval

Ten of the RTC studies [3, 15, 17–24] reported the functional knee society score, and the results were analyzed at the final assessment. The final assessment ranged from 1 to 3 years in seven studies, 5 years in one study, and 10 years in two studies. None of the papers reported a statistically significant difference between APT and MBT tibias, and our analysis confirmed this. Figure 3 displays the forest plot of the functional knee society score, which shows no statistical difference between the two tibial designs (OR 1.38; 95% CI − 0.47–3.23, *p* value 0.14) and high heterogeneity across the results (I2 = 84%).

### Clinical knee society score

**Fig. 4:**
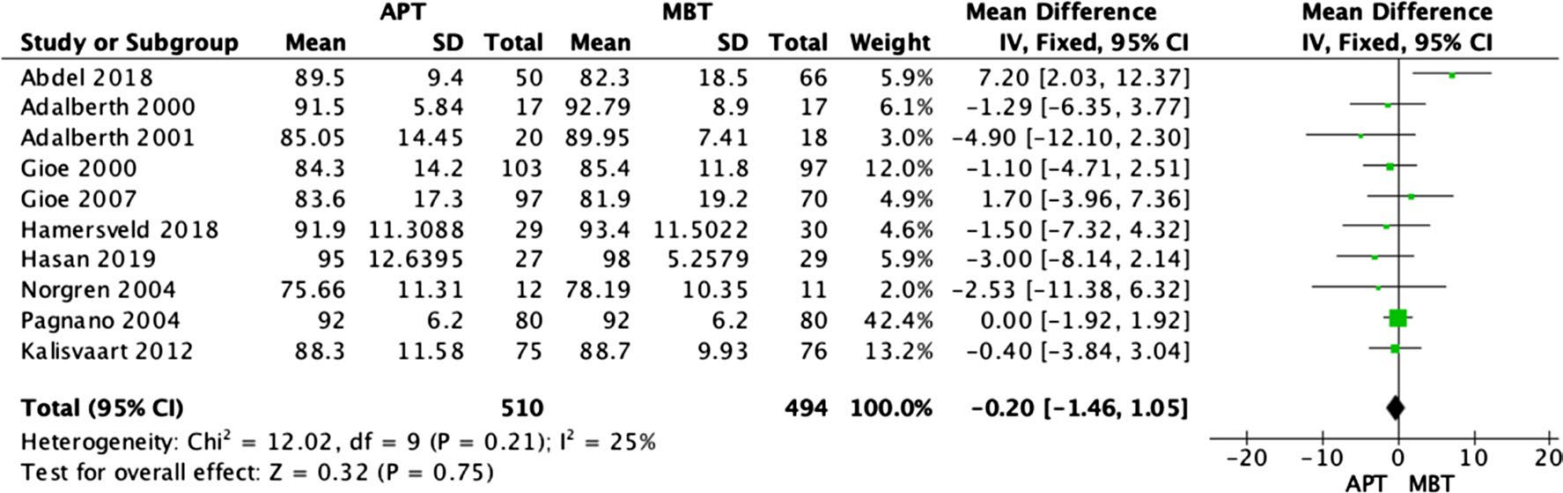
Forest plot of clinical knee society score at the final follow-up between all-polyethylene (APT) and metal-backed (MBT) tibias, CI: confidence interval

Ten RCTs [3, 15, 17–24] reported the clinical or pain knee society score, and none of them reported a statistically significant difference at the final follow-up, which ranged from 1 to 10 years. This was also confirmed by the meta-analysis (OR − 0.20; 95% CI − 1.46–1.05, *p* value 0.75). Figure 4 presents the forest plot of the pooled data from the included papers, and it shows no statistically significant difference between the two groups (*p* = 0.75). The heterogeneity was low, with an I2 value of 25%.

### Knee range of motion

**Fig. 5:**
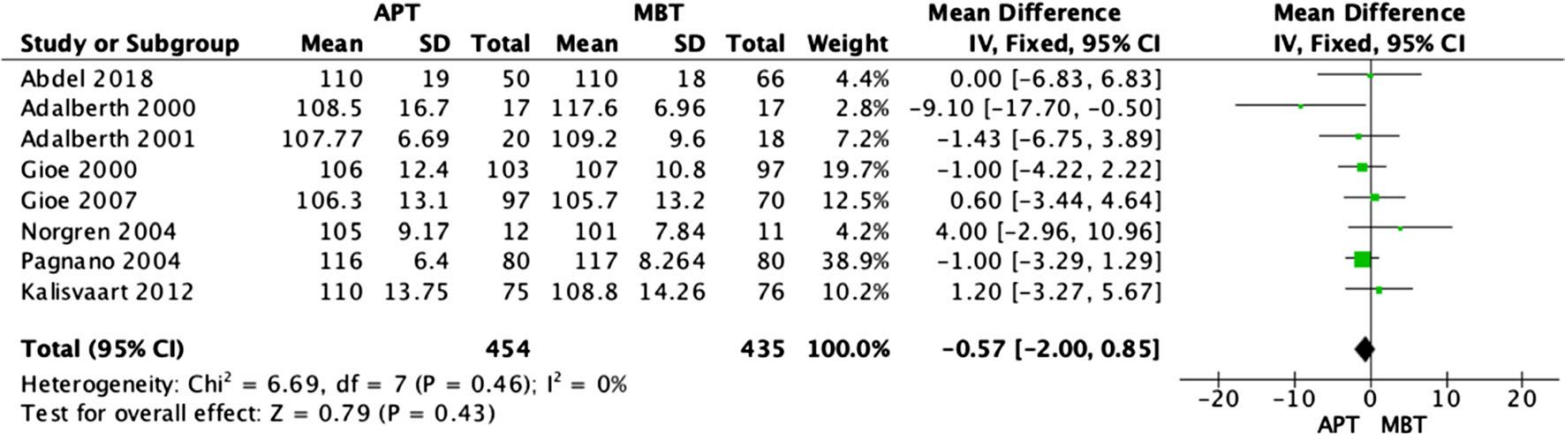
Forest plot of degree of knee flexion at the final follow-up between all-polyethylene (APT) and metal-backed (MBT) tibias, CI: confidence interval

At the final follow-up, eight studies [3, 17–19, 21–24] reported the degree of knee flexion among APT and MBT designs. The fixed effect of the meta-analysis revealed no statistically significant difference between the two groups, as demonstrated in Fig. 5 (OR − 0.57; 95% CI − 2.00–0.85, *p* value 0.43).

### Stairs climbing score

**Fig. 6:**
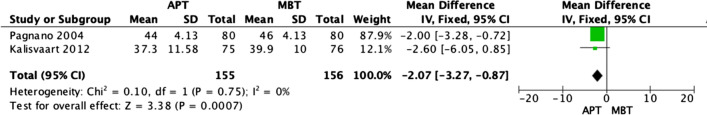
Forest plot of the stairs climbing score at the final follow-up between all-polyethylene (APT) and metal-backed (MBT) tibias, CI: confidence interval

The stairs climbing score was reported in both Pagnano et al. [24] and Kalisvaart et al. [18], with a total of 311 patients randomized between the two tibial designs at the final follow-ups of 1 year and 5 years, respectively. According to our fixed model analysis, the all-polyethylene tibia was statistically superior in the stairs climbing score at the final follow-up of the included studies. This is shown in Fig. 6 (OR − 2.07; 95% CI − 3.27 to − 0.87; *p* value 0.0007).

### Radiostereographic analysis (RSA)

**Fig. 7:**
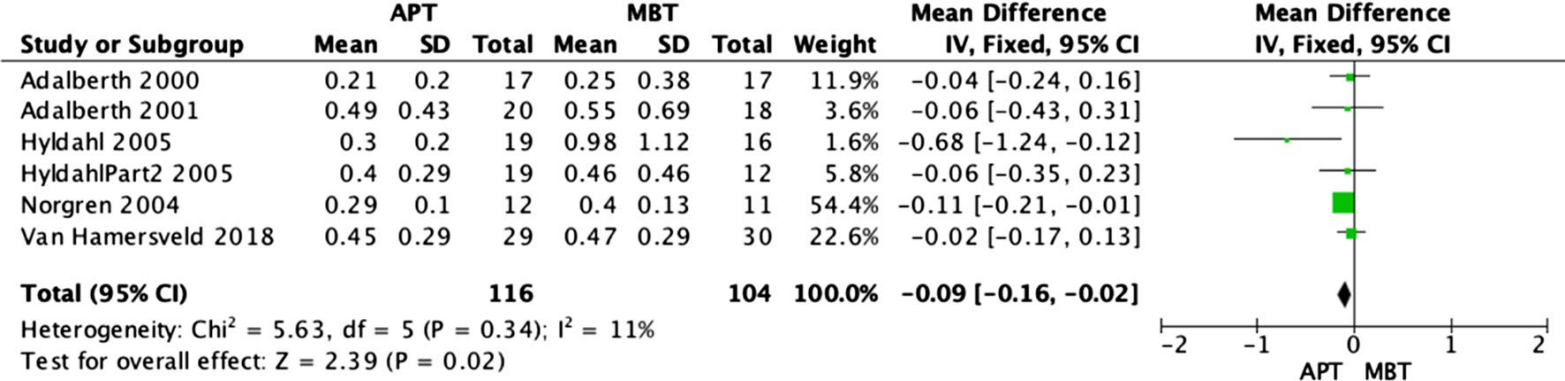
Forest plot of RSA AP rotation at 2 years between all-polyethylene (APT) and metal-backed (MBT) tibias, CI: confidence interval

**Fig. 8:**
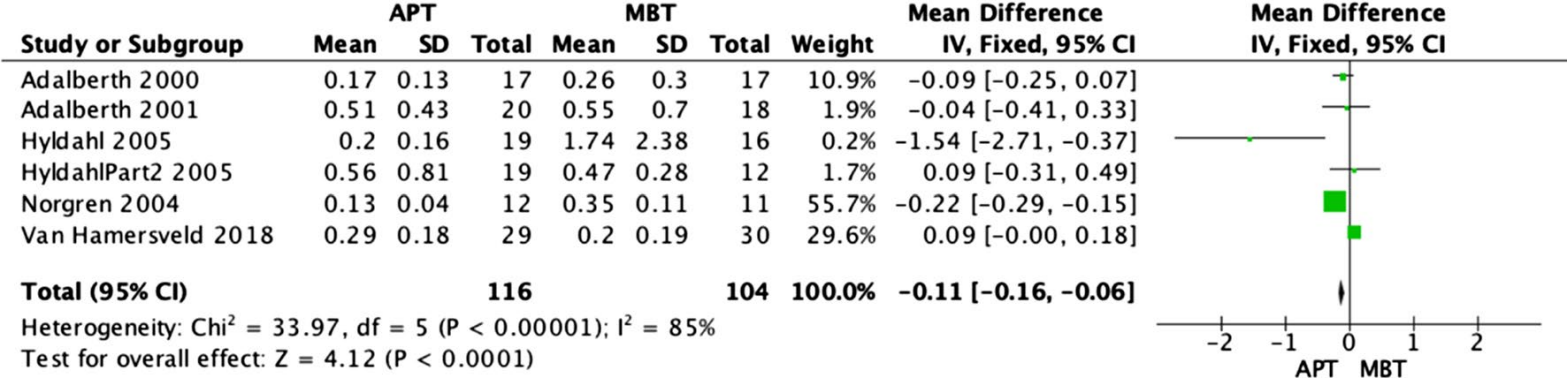
Forest plot of RSA internal–external rotation at 2 years between All-polyethylene (APT) and metal-backed (MBT) tibias, CI: confidence interval

**Fig. 9:**
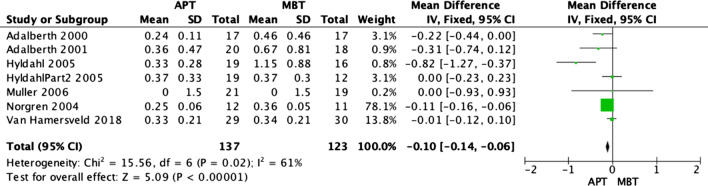
Forest plot of RSA varus–valgus malalignment at 2 years between all-polyethylene (APT) and metal-backed (MBT) tibias, CI: confidence interval

**Fig. 10:**
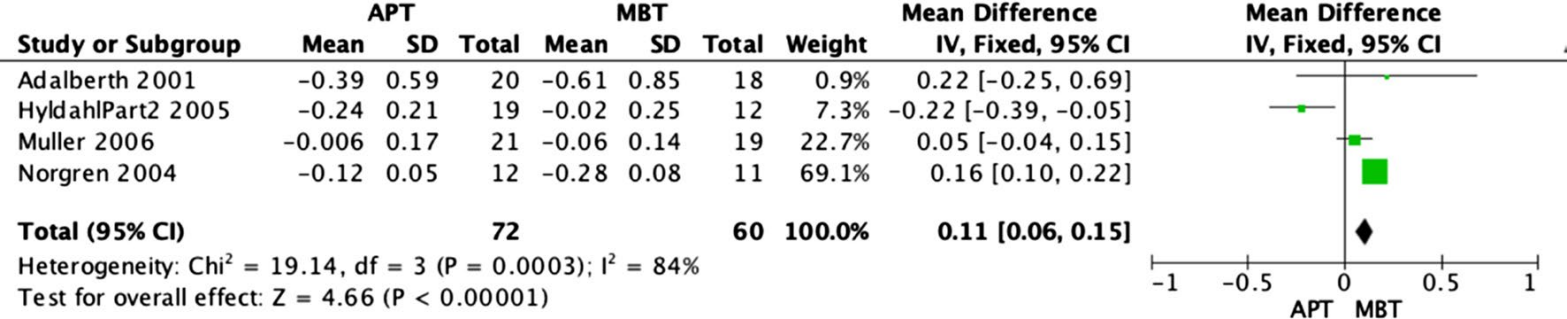
Forest plot of RSA maximum implant subsidence at 2 years between all-polyethylene (APT) and metal-backed (MBT) tibias, CI: confidence interval

Radiostereographic analysis was performed in 7 studies [3, 15, 19, 21, 25, 26] of the analyzed RCTs, with all of these papers reporting RSA at 2 years. None of these studies showed any significant implant migration differences between APT and MBT designs. However, our fixed model analyses revealed that MBT knees were significantly superior in terms of anterior–posterior rotation (Fig. 7) and internal–external rotation migration (Fig. 8) at 2 years, as shown in the data pooled from 6 studies [3, 15, 19, 21, 25] with (OR − 0.09; 95% CI − 0.16 to − 0.02; *p *value 0.02) and (OR − 0.11; 95% CI − 0.16 to − 0.06, *p* value < 0.0001), respectively. Moreover, MBT knees were also superior in terms of varus–valgus malalignment (Fig. 9) at 2 years, as shown in the data pooled from 7 studies [3, 15, 19, 21, 25, 26] (OR − 0.10; 95% CI − 0.14–0.06, *p* value < 0.00001). Additionally, all-polyethylene knees were significantly higher rates in terms of maximal implant subsidence at 2 years (OR 0.11; 95% CI 0.06–0.15, *p* value < 0.00001), as shown in Fig. 10 [3, 19, 25, 26]. Although APT appeared to be more stable statistically, it is unknown whether these results are clinically significant.

### Complication rate

**Fig. 11:**
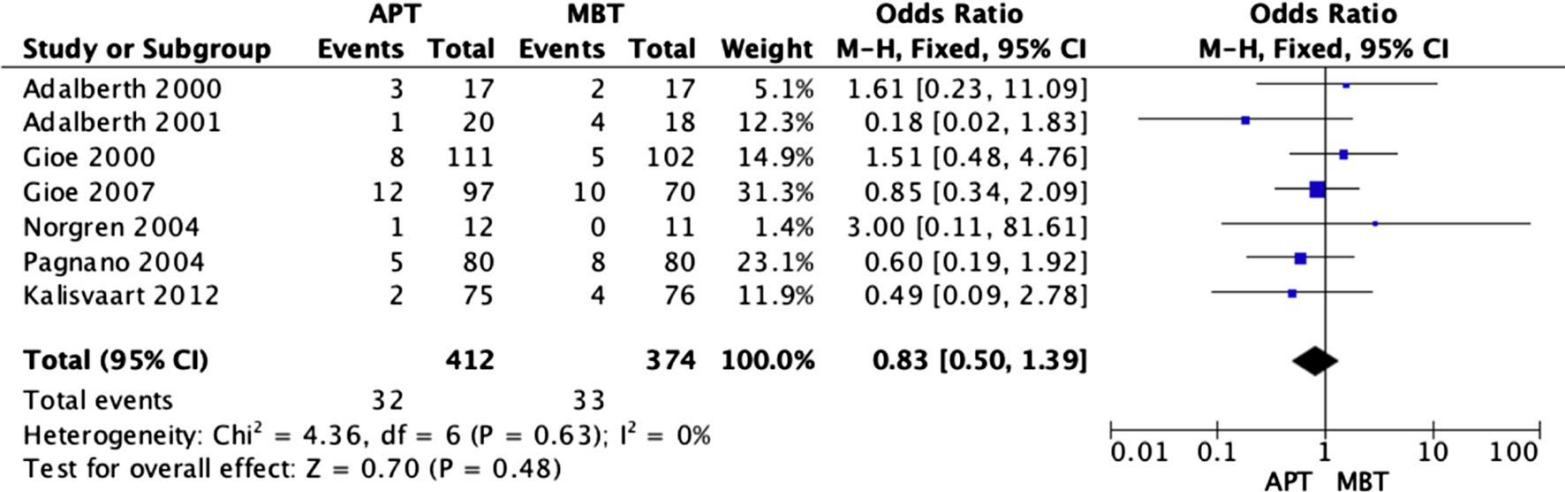
Forest plot of complication rate at the final follow-up between all-polyethylene (APT) and metal-backed (MBT) tibias, CI: confidence interval

Seven RCTs [3, 18, 19, 21–24] reported on complication events at their final follow-ups, which ranged from 1 to 10 years. While more events occurred in the MBT group, the overall fixed model analysis showed no significant difference (OR 0.83; 95% CI 0.5–1.39, *p* value 0.48). Figure 11 displays the forest plot of the pooled data. Pooled complications were mainly infection, aseptic loosening and knee stiffness.

### Survivorship

**Fig. 12:**
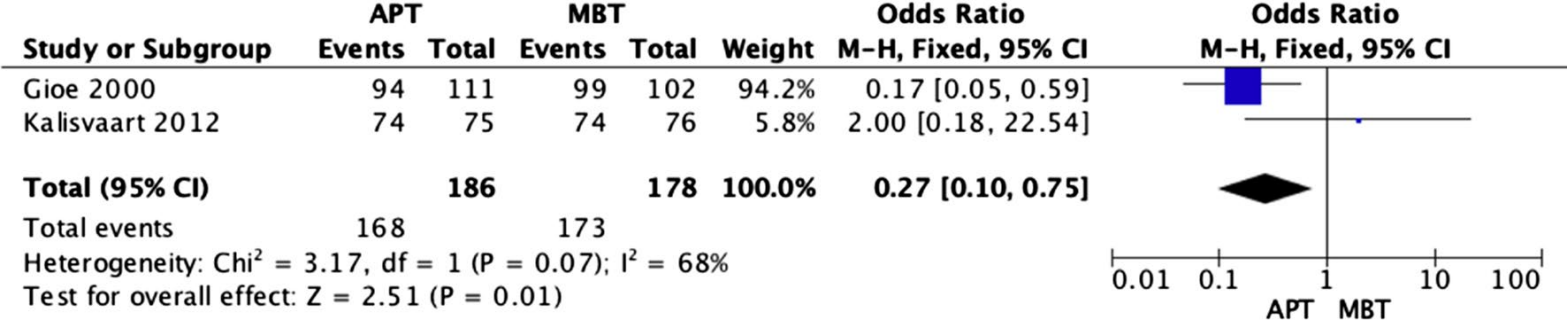
Forest plot of implant survivorship at 5 years between all-polyethylene (APT) and metal-backed (MBT) tibias, CI: confidence interval

**Fig. 13:**
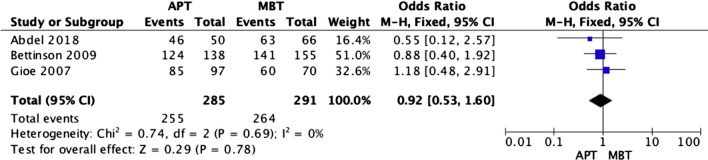
Forest plot of implant survivorship at 10 years between all-polyethylene (APT) and metal-backed (MBT) tibias, CI: confidence interval

Survivorship percentages were reported in the included studies. Data on two endpoints were pooled, with two studies [18, 23] reporting implant survivorship at 5 years and three others [16, 17, 22] reporting at 10 years. At 5 years, the all-polyethylene tibia design showed a lower revision rate and appeared to be superior compared to the metal-backed counterpart (OR 0.27; 95% CI 0.10–0.75; *p* value 0.01) (Fig. 12). However, there was no significant difference between the survivorship of both designs at 10 years (OR 0.92; 95% CI 0.53–1.60; *p* value 0.78) (Fig. 13). It was not possible to pool the type of polyethylene used in the studies or whether they were treated with vitamin E or not. Also, no study has reported results beyond 10 years.

## Discussion

Despite the relative lack of popularity compared to metal-backed counterparts in current practice, the literature suggests that all-polyethylene tibial trays yield comparable results. The most significant findings of this meta-analysis were that the all-polyethylene tibial tray was associated with better outcomes, including higher scores on stair climbing, short-term survivorship, and maximal implant subsidence. Furthermore, this study found that all of the randomized trials included in our analysis showed comparable results in terms of overall complication rates, long-term survivorship, range of motion and knee society scores.

The selection of tibial components for total knee arthroplasty has been a topic of debate throughout the development of the procedure. While surgeons from various backgrounds may claim the effectiveness of one implant over another, randomized trials have failed to demonstrate the superiority of any particular implant [14, 27–29]. For example, studies have shown that fixed-bearing versus mobile-bearing tibias, cruciate-retaining versus posterior-stabilized, and metal-backed versus all-polyethylene tibias all yield similar outcomes [30].

Over the past decade, several meta-analyses have been conducted to compare the two tibial designs [31–36]. However, apart from the study by Tao Cheng et al. in 2011, none of the other studies were classified as Level I evidence [35]. While the results of most studies showed comparable outcomes for both tibial designs, authors often cautioned readers to interpret their conclusions carefully due to publication bias and poor methodological quality of the included studies. Cheng et al. [35] published the only Level I meta-analysis, which included data from nine randomized controlled trials (RCTs) and only pooled complication rates using a fixed model. While the authors conducted a systematic review of functional knee scores, quality of life scores, range of motion, and implant position for each RCT, none of these data were systematically analyzed in fixed or random effect models. In contrast, our study included 14 RCTs, and all of our results were presented in a systematic analysis pattern, where studies with similar outcomes were pooled together and analyzed.

When considering the similar clinical performance of both tibial designs, cost becomes an important factor. While some studies have reported cost savings of up to 30% with the all-polyethylene tibia [3, 4], others have claimed the opposite [14, 32]. Longo et al. [32], in their Level III meta-analysis in 2016, found that metal-backed and all-polyethylene tibias had comparable clinical outcomes and range of motion. However, they also found that complications and revision rates were higher with the all-polyethylene design, making metal-backed tibias more cost-effective. Despite analyzing 32 studies, there was significant heterogeneity among the included studies in the later meta-analysis [32], and there were inadequate reports on the complications. In addition to the issues with heterogeneity and inadequate reporting of complications in the Longo et al. meta-analysis [32], some of the studies included were reporting on older versions of polyethylene that have since lost popularity due to wear and weaker mechanical characteristics. This may explain their reporting of a higher complication rate with all-polyethylene tibias. In contrast, our study found fewer complications and a lower early revision rate when more contemporary polyethylene components were studied. This could be attributed to the recent use of vitamin E-treated polyethylene, which has been shown to have theoretical superiority in vitro, although not necessarily in vivo [37, 38].

Our study also included radiostereographic analysis (RSA) as a means of comparing the two tibial designs. Interestingly, we found that the metal-backed design was significantly more radiographically stable in anterior–posterior, varus–valgus, and internal–external rotations at the 2-year follow-up compared to all-polyethylene tibias. However, caution should be exercised when interpreting these results due to significant heterogeneity among the studies. Moreover, the metal-backed design had significantly less maximal implant subsidence. This is consistent with the findings of Nouta et al. [34], who conducted a systematic review in 2012 and similarly reported better implant fixation in all-polyethylene tibias with lower maximum total point motion compared to the metal-backed counterpart.

Our study's main advantage is its inclusion of high-quality articles in the meta-analysis, making it, to our knowledge, the only level 1 evidence meta-analysis on the subject. We analyzed all 14 RCTs comparing the two tibial design concepts. However, the heterogeneity of the studies limited the sample size we could analyze, and some studies reported outcome measures that others did not. We also encountered difficulties reporting outcomes at the last follow-up, as the studies had different follow-up periods, so we reported our results at the final follow-up assessment instead of a mean time with a wide range of final surveillance.

## Conclusion

While this meta-analysis suggests that all-polyethylene tibial components in total knee arthroplasty may offer advantages over metal-backed components in terms of five-year survivorship, and stairs climbing score, this finding should be considered in the context of potential confounding factors. Nonetheless, based on the results, the all-polyethylene implant should be considered a viable choice for primary knee replacement.

## Data Availability

Not applicable as this is a review article. However, happy to provide access to any statistical data (coding) upon request.
